# Phyllanthus amarus protects against spatial memory impairment induced by lipopolysaccharide in mice

**DOI:** 10.6026/97320630015535

**Published:** 2019-08-31

**Authors:** Akilandeshwari Alagan, Ibrahim Jantan, Endang Kumolosasi, Norazrina Azmi

**Affiliations:** 1 Drug and Herbal Research Centre,Faculty of Pharmacy,Universiti Kebangsaan Malaysia, Jalan Raja Muda Abdul Aziz,50300 Kuala Lumpur,Malaysia; 2 School of Pharmacy-SRI,Faculty of Health and Medical Sciences,Taylor's University,Lakeside Campus,Jalan Taylor's,47500 Subang Jaya, Selangor, Malaysia

**Keywords:** Phyllanthus amarus, spatial memory, neuroprotection, neuroinflammation, lipopolysaccharide

## Abstract

Phyllanthus amarus Schumach. and Thonn. is a wide spread medicinal herb with various traditional uses. It is well documented for its antioxidant, anti-inflammatory, and hepatoprotective activities. Therefore, it is of interest to evaluate the 80% ethanol extract of Phyllanthus amarus (PA) on spatial memory using the 8-radial arm maze (8-RAM) in mice after induction of neuro inflammation by lipopolysaccharide (LPS) in a 14- and 28-days treatment study. LC-MS/MS was performed to profile the chemical composition in PA extract. Mice were treated orally with 5% v/v tween 20, PA extract (100, 200 and 400 mg/kg), or ibuprofen (IBF 40 mg/kg) for 14 and 28 days. All groups were challenged with LPS (1 mg/kg) via intraperitoneal (i.p.) injection a day prior to the 8-RAM task except for the negative control group which received an i.p. injection of saline. Data obtained were analyzed with one-way ANOVA followed by post hoc Dunnett's test (comparison of all groups against vehicle control). Analysis of LC-MS/MS data revealed the presence of 16 compounds in the PA extract. Administration of PA extract at 200 and 400 mg/kg for 14 and 28 days significantly (*P<0.05) decreased the working and reference memory errors against LPS-induced spatial memory impairment. The observed protective action is possibly due to the putative antineuroinflammatory effects of PA. In conclusion, PA extract possess neuroprotective effects against spatial memory impairment mediated by LPS.

## Background

Phyllanthus amarus Schumach. and Thonn. is a medicinal herb which is widely distributed in tropical and subtropical regions. Traditionally it has been used for fever, diarrhea, colic, diuresis and kidney aliments [1]. Several studies reported the presence of various compounds in the plant including alkaloids, flavonoids, lignans, ellagitannis, triterpenes, polyphenols, tannins, sterols and volatile oils [2]. Previous studies in our laboratory have identified the presence of phyllanthin, hypophyllanthin, gallic acid, geraniin, corilagin, ellagic acid, and niranthin in Phyllanthus amarus (PA) extract [3]. PA is well documented for its anti-oxidant and antiinflammatory activities. Administration of the aqueous extract of PA effectively reversed the amnesia induced by scopolamine and has been traditionally used for nervous debility [4]. Other species of Phyllanthus like P. emblica improved learning and memory using a battery of cognitive-behavioral tests such as Morris water maze, elevated plus maze, passive avoidance and rewarded alternation test [5,6]. Similarly, administration of P. reticulatus in rats administered with aluminium resulted in enhanced memory in passive avoidance and rewarded alternation test [7]. P. niruri enhanced motor and neuromuscular coordination in rats [8] and P. acidus was also proven to ameliorate the spatial long-term and recognition memory in rats [9,10]. Treatment with PA extract and phyllanthin were found to improve memory impairment and exhibited anti-cholinesterase activity in young and older mice [4,11]. In addition, we have demonstrated the protective effects of PA against non-spatial memory impairment mediated by neuro inflammation [12]. However, studies on the effects of PA on different types of memory are scarce. Therefore, the present study sought to investigate the protective effects of a chemically characterized PA extract on spatial memory impairment induced by LPS in mice. This study is an extension of our recent work as described earlier [12].

In the last 60 years, understanding the nature of amnestic disorder has extended immensely. The improved understanding was not only generated from the growing body of knowledge on memory disorders affecting humans, but also from various animal studies performed in different strains, particularly in memory deficits. These animal models have always been a reflection of amnesic disorder in humans, which helped us to understand memory dysfunction [13]. Memory is the process of gaining information from the surroundings and consolidating the attained information and reclaim for future purpose. There are different types of assessment for testing memory process (long term and short-term memory). In cognitive function, spatial discrimination is considered as an important task, which is shared among wide species including honeybees to humans [13,14]. Radial arm maze (RAM) is one of the sensitive tasks in spatial memory assessment. Successful performance begins by utilizing the spatial map and extra maze cues. RAM is effective in obtaining two types of information (spatial versus intra maze cues) and memory functions (reference memory and working memory). It is designed either as an 8 or a 12 arms maze with the central compartment, particular arms are regarded as spatial working and reference memory arms. When rodents frequently visit the arm that never had food reward is regarded as reference memory or long-term memory error whereas the re-entry of rodents into the rewarded arms is known as working memory or short-term memory error [15].

A plethora of studies documented that the connection between working and reference memory lies within the brain structures. For example, hippocampus and other limbic structures (septum and amygdala) are suggested as the most important parts involved in the memory function especially in RAM. Therefore, the lesion of these particular regions resulted in the inaccuracy of memory function in RAM [14]. Peripheral injection of bacterial endotoxin (lipopolysaccharide, LPS) can result in sickness behaviour through activation of microglia cells, which induce pro-inflammatory mediators in the brain [16]. A single systemic injection of LPS can activate the immune system and impairs spatial memory although it is an acute effect [16]. Moreover, repeated exposure to LPS leads to chronic defects in the brain [16,17]. Therefore, in this study, we evaluated the effects of 14- and 28-days treatment of 80% ethanol extract of PA against LPS-induced spatial memory impairment in mice.

## Subjects and Methods:

### Animals:

Adult male ICR mice weighing between 25-30 g (5 weeks old) were obtained from the Laboratory Animal Resource Unit (LARU), Universiti Kebangsaan Malaysia (UKM), Malaysia. The mice were housed in the temperature-controlled room (22-25 °C), exposed to 12 h dark/light cycles and allowed to access free food. Experiments were carried out by following the standard protocol approved by UKM Animal Ethical Committee with the approval number FF/2016/NORAZRINA/27-JULY/774-JULY-2016-JULY- 2017. Experiments were started after 5 days of acclimatization to the laboratory environment.

### Plant materials:

The entire plant of Phyllanthus amarus Schumach. and Thonn. was acquired from Marang, Kuala Terengganu, Malaysia. Dr. Abdul Latif Mohamad from the Faculty of Science and Technology, Universiti Kebangsaan Malaysia (UKM) confirmed the plant and the voucher samples (P. amarus UKM 30078) was deposited at the Herbarium of UKM, Bangi, Malaysia.

### Preparation of extract:

After gathering the plant, it was allowed to dry for a week and the coarse powder was granulated. A 1 kg of coarse powder was soaked in 80% ethanol for 9 days. The substance was filtered, and the solvent was changed every three days. The solvent was evacuated utilizing rotary evaporator, at that point, the concentrate was subjected to freeze-drying and stored in - 20°C.

### Liquid chromatography-tandem mass spectroscopy analysis:

Liquid chromatography-tandem mass spectroscopy (LC-MS/MS) for PA was analyzed by Perkin Elmer Flexar FX15 UHPLC system coupled to Sciex 3200 hybrid trap triple quad tandem mass spectrometer (UHPLC-MSMS). Phenomenex Synergy RP C18, 100A (100 mm x 2 um x 2.0 mm) column was used. Gradient elution technique was carried out at 1 mL/min using water (0.1% formic acid) as solvent A and acetonitrile (0.1%) formic acid as solvent B, run for 70 min, 20µL of sample injection. The gradient elution started at 5% B (0-3 min); 80% B (3-10 min); 80% B (10-15 min) and 5% B (15-22 min). The positive and negative ionization spectra were acquired with MicroTOF QIII Bruker Daltonic with the following parameters: - capillary voltage: 4500 V; nebulizer pressure: 1.2 bar; drying gas: 8 L/min at 200°C. The mass range was at 50-1000 m/z. The accurate mass data of molecular ions were processed by Compass Data Analysis software (Bruker Daltonik GmbH). A mass spectral library was used to identify the corresponding peaks of the compounds.

## Test groups:

### LPS optimal dose determination:

A pilot study was carried out in a total of three groups of mice where n=5 in each group. Group 1 was regarded as vehicle control (0.9% normal saline), group 2 and 3 received 0.5 and 1 mg/kg LPS via i.p. respectively. A day after LPS induction, the test phase was performed to analyze the working and reference memory errors.

### Effects of PA extract on spatial memory impairment induced by LPS:

The 14 and 28 days treatment was carried out in a total of six groups of male ICR mice where n=8 in each group. The negative and vehicle control groups were administered 5% v/v tween 20 orally and saline via i.p., 40 mg/kg of ibuprofen p.o., as positive control and PA extract given at 100, 200 and 400 mg/kg respectively. All groups were challenged with 1 mg/kg of LPS via i.p. on day 13th and 27th except the negative control group and the next day the rats were subjected to behavioral task.

### The 8-Radial Arm Maze (RAM):

The 8-RAM is widely used to assess working memory and reference memory of mice. The 8-RAM procedure was conducted based on methods reported by Hritcu and Nabeshima and Tarragon et al., with minor modifications as described below [18,19].

### Behavioral apparatus:

It consists of eight arms lengthening radially from a central region with 32 cm in diameter, made up of Perspex. The apparatus was bounded by visual clues and positioned at 40 cm above the floor. Food pellet was placed at the edge of arms. The 8-RAM was placed inside the behavioral room with continuous illumination of 35W yellow halogen light and connected to CCTV footage. Stop watch was used to keep the time.

## Procedure:

One day prior to the experiment, mice were deprived of food. The test consisted of three phases: habituation, training and test phase. During the habituation phase, 8 arms were opened. Each mouse was freely allowed to visit the arms in the duration of 5 min for three days and they were returned to their respective home cages. After habituation, rodents were allowed one trail per day for eight days. In the training phase, among eight arms, four randomly selected arms were baited, and another four arms were closed and not baited. Followed by the test phase, the rodents were allowed to access the alternative baited arm for 5 min. The experiment was continued until the food was consumed by the rats or until 5 min duration. In between each trial, 70% of ethanol solution was used to clean the apparatus to eliminate the olfactory cues. The number of entry into each arm was scored if the rodents entered the arm with all four limbs. Working and reference memory errors were scored on a replay of the CCTV footage with the experimenter blinded to the treatment group. The number of re-entry into the baited arm was regarded as working memory error. Whereas the number of entries into unbaited arm in the test phase was interpreted as reference memory error.

## Statistical Analysis:

Data obtained was analyzed with one-way ANOVA followed by Post hoc Dunnett's test (comparison of all groups against vehicle control) using GraphPad Prism 5. The data were presented as mean ± standard error of the mean (SEM) with n=8. P < 0.05 value was set as statistically significant.

## Results:

### Liquid chromatography-tandem mass spectroscopy analysis:

**Figure 1:**
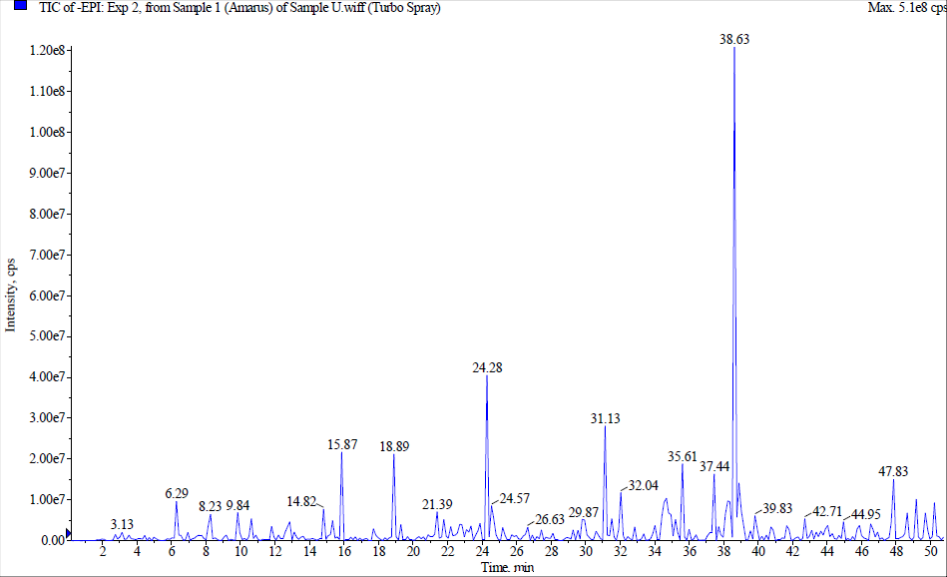
LCMS/MS chromatogram of P. amarus extract

Analysis of phenolics and lignans was performed using HPLC, GC and GC/MS, but in recent time, LC/MS has been widely used to detect the compounds in research [20]. LC/MS is widely used because of its speed, sensitivity, specificity and coupling ability with chromatographic techniques [21]. Thus, to identify the tentative compounds in PA extract, LCMS/MS technique was performed. The phytoconstituents were identified based on their retention time, UV spectra and mass fragmentation (Figure 1). The data from PA extract revealed 16 compounds in negative ionization mode (Table 1).Hydrobenzoic acid, flavonols, ellagic acid derivatives were detected in negative mode. The results were similar to that of Kumar and colleagues [22]. In addition, a previous study performed by our colleagues has similarly identified phyllanthin, hypophyllanthin, gallic acid, geraniin, corilagin, ellagic acid, niranthin, phyltetraline, and isonilteraline in the PA extract using high-performance liquid chromatography [3].

### Determination of optimal dose for the induction of memory deficits in mice:

**Figure 2:**
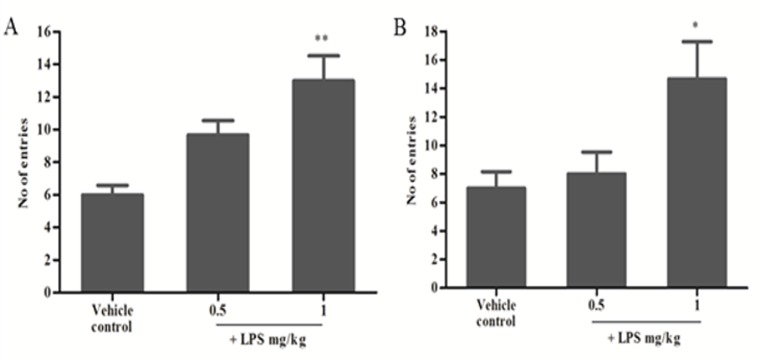
Determination of LPS dose in 8-Radial arm maze-(A) Working memory error (B) Reference memory. Data (n=8) represent the mean time (± SEM). **p<0.01, and *p<0.05 vs Vehicle control using one-way analysis ANOVA followed by Post hoc Dunnett's test. LPS-Lipopolysaccharide.

The frequency of working and reference memory errors in the vehicle control group showed lesser error than LPS-induced groups. LPS administration at different doses of 0.5 and 1 mg/kg showed increased working and reference memory errors. Among the tested two doses, 1 mg/kg administration of LPS showed a significant increase (**P<0.01, *P<0.05) of working and reference memory errors which imply that the mice failed to recall their memory or ignored in the particular phase or mistakenly chose the unbaited arm as the rewarded arm (Figure 2 A and B ).

### Effects of PA extract on spatial memory impairment induced by LPS:

**Figure 3:**
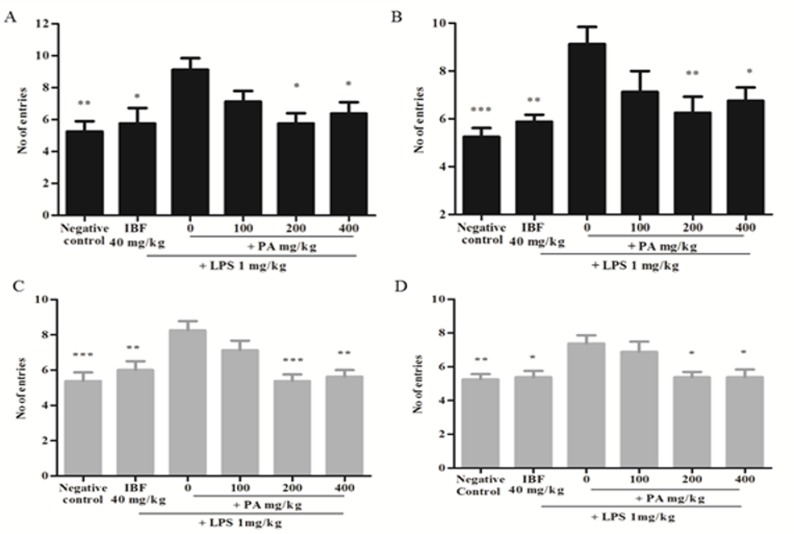
Errors in working and reference memory after 14 (A and B) and 28 (C and D) days of PA extract treatment. Data (n=8) represent the mean time (± SEM). (*P<0.05, **P<0.01, ***P<0.001 vs LPS respectively using one-way analysis ANOVA followed by Post hoc Dunnett's test. LPS-Lipopolysaccharide, IBF- Ibuprofen, and PA- Phyllanthus amarus extract.

After 14 days of treatment with PA extract at 200 and 400 mg/kg (**P<0.01, *P<0.05), a significant reduction in working and reference memory errors was observed when compared to vehicle control (Figure 3 A and B). Similarly, 28 days treatment of 200 and 400 mg/kg PA extract showed lesser error (*P<0.05, **P<0.01, ***P<0.001) when compared to vehicle control (Figure 3 C and D ). Negative control and IBF groups showed a significant (*P<0.05, **P<0.01, ***P<0.001) decrease in errors against the vehicle control group. However, 100 mg/kg of PA extract administered for 14 and 28 days could not reverse the memory impairment mediated by LPS as compared to vehicle control.

## Discussion:

Many medicinal plants have been widely documented for improving cognitive functions such as Centella asiatica, Withania somnifera, and Gingko biloba [23]. This pharmacological activity of the medicinal plants is due to the presence of bioactive phytochemicals. Based on previous evidence of Phyllanthus species on memory improvement [4-11], PA extract was investigated for protection against spatial memory impairment induced by LPS after 14- and 28-days of oral administration. Preliminary identification of phytoconstituents in PA extract by LC-MSMS revealed the presence of tentative phytoconstituents such as brevifolin acid, brevifolin carboxylic acid isomer, ethyl gallate, strictinin, gallic acid, geraniin, benzenoid compound, hyperin, rutin, quercetin-3-glucuronide, kaempferol monosulfate, 3,30-di-Omethyl ellagic acid, caffeic acid, and 2(3,4-dihydroxyphenyl)-7- hydroxy-5-benzene propanoic acid. Phytoconstituents such as gallic acid, geraniin, and rutin were reported to possess neuroprotective properties by improving the behavioral score and inhibiting proinflammatory cytokines, and β-secretase in models of neuroinflammation [24-26].

The 8-RAM is a successful method to assess whether a test compound produces improvement or deleterious effects on cognition [27]. A single i.p. Injection of LPS in mice induced more spatial memory deficits at a higher dose of 1 mg/kg than the lower dose of 0.5 mg/kg. It denotes that mice were unable to recall former information while searching for the food in the arms. Similarly, previous studies have demonstrated an increase number of working and reference memory errors in LPS-induced rats [16]. Disruption of learning and memory in rodents may be due to the activation of the immune system in LPS-induced groups, which led to cytokine production and resulted in cognitive deficits [11,16]. Thus, the determined dose of LPS from the pilot study was used to induce spatial memory impairment for the subsequent study to determine the protective effects of PA extract.

In this navigation task, PA treated groups showed a decrease in working and reference memory errors compared to the vehicle control group after 14- and 28-days of pre-treatment. Our results are in concordant with the effects of other Phyllanthus species such as P. emblica, P. reticulatus, P. niruri, and P. acidus, which were reported to improve spatial memory in animals, challenged with various inducers of memory impairment [4-9]. This improvement in cognition was suggested to be due to the reduction of oxidative stress, which increases brain anti-oxidant enzymes level [4-9]. Our previous study using the same PA extract given orally for 14 and 28 days has shown that the extract was able to protect against nonspatial memory deficits in rats, which resulted from inflammatory processes in the brain [12]. Indeed, the observed neuroprotective effects were possibly due to the suppression of inflammatory mediators like TNF-α, IL-1β, iNOS, NO, CD11b/c and an increased in synaptophysin marker through the inhibition of toll-like receptor 4 (TLR4) expression in LPS-induced rats [12]. In the present study, PA protected against impairment of spatial memory mediated by LPS in mice. Therefore, it becomes increasingly evident that PA has neuroprotective actions on different types of memory. We suggest that the protective effects of PA on spatial memory may be mediated by alleviation of immunological responses in the brain but further studies are warranted to proof the concept.

## Ethics approval and consent to participate:

Experiments were carried out by following the standard protocol approved by the UKM Animal Ethical Committee with the approval number FF/2016/NORAZRINA/27-JULY/774-JULY- 2016-JULY-2017

## Figures and Tables

**Table 1:** LCMS/MS- Tentative compounds in PA extract

Retention time	Molecular ion	m/z fragmentation ion	Tentative compounds
(RT in min)	peak (M-H)-		
8.25	247.18	247.02*, 219.06, 190.00	Brevifolin acid
9.83	291.084	291.01*, 247.02, 219.08, 190.99	Brevifolin carboxylic acid isomer
14.82	197.16	197.03*, 168.99, 140.02, 124.96	Ethyl gallate
15.87	633.02	633.02*, 462.98, 301.00, 274.96	Strictinin
17.7	466.02	465.91*, 301.03, 229.02, 168.99, 124.97, 95.10	Gallic acid
18.89	950.88	950.95*, 932.99, 300.97, 272.99	Geraniin
21.78	272.96	272.99*, 245.00, 217.01, 189.01, 161.00, 145.00	Benzenoid compound
22.57	463.11	463.03*, 316.02, 299.94	Hyperin
24.28	609.13	609.12*, 300.00, 271.02	Rutin
24.54	477.06	477.02*, 301.02, 255.13, 179.02, 150.99	Quercetin-3-glucuronide
31.52	364.94	365.01*, 350.01, 230.93, 151.01	Kaempferol monosulfate
32.83	329.07	328.99*, 313.98, 298.98, 270.99, 242.92	3,30-di-O-methyl ellagic acid
43.76	447.22	447.17*, 315.30, 179.08	Caffeic acid
57.492	311.08	311.40*, 239.36, 183.23, 119.01	2(3,4-Dihydroxyphenyl)-7-hydroxy-5-benzene propanoic acid
